# Presentation, Treatment Response and Short-Term Outcomes in Paediatric Multisystem Inflammatory Syndrome Temporally Associated with SARS-CoV-2 (PIMS-TS)

**DOI:** 10.3390/jcm9103293

**Published:** 2020-10-14

**Authors:** Susanna Felsenstein, Emily Willis, Hannah Lythgoe, Liza McCann, Andrew Cleary, Kamran Mahmood, David Porter, Jessica Jones, Janet McDonagh, Alice Chieng, Giulia Varnier, Stephen Hughes, Mary Boullier, Fiona Ryan, Olumoyin Awogbemi, Giridhar Soda, Phuoc Duong, Clare Pain, Phil Riley, Christian M. Hedrich

**Affiliations:** 1 Department of Infectious Diseases and Immunology, Alder Hey Children’s NHS Foundation Trust Hospital, Liverpool L14 5AB, UK; David.Porter@alderhey.nhs.uk; 2 Department of Rheumatology, Royal Manchester Children’s Hospital, Manchester M13 9WL, UK; Emily.Willis@mft.nhs.uk (E.W.); janet.mcdonagh@manchester.ac.uk (J.M.); Alice.Chieng@mft.nhs.uk (A.C.); giuliavarnier@gmail.com (G.V.); stephen.hughes@mft.nhs.uk (S.H.); Phil.Riley@mft.nhs.uk (P.R.); 3 Department of Rheumatology, Alder Hey Children’s NHS Foundation Trust Hospital, Liverpool L14 5AB, UK; Hanna.Lythgoe@alderhey.nhs.uk (H.L.); Liza.McCann@alderhey.nhs.uk (L.M.); Gavin.Cleary@alderhey.nhs.uk (A.C.); Kamran.Mahmood@alderhey.nhs.uk (K.M.); Clare.Pain@alderhey.nhs.uk (C.P.); 4 Department of Microbiology, Alder Hey Children’s NHS Foundation Trust Hospital, Liverpool L14 5AB, UK; jessica.jones@alderhey.nhs.uk; 5 Department of General Paediatrics, Alder Hey Children’s NHS Foundation Trust Hospital, Liverpool L14 5AB, UK; mary.boullier@alderhey.nhs.uk (M.B.); fiona.ryan@alderhey.nhs.uk (F.R.); olumoyin.awogbemi@alderhey.nhs.uk (O.A.); 6 Department of Cardiology, Royal Manchester Children’s Hospital, Manchester M13 9WL, UK; giridhar.soda@alderhey.nhs.uk; 7 Department of Cardiology, Alder Hey Children’s NHS Foundation Trust Hospital, Liverpool L14 5AB, UK; Phuoc.Duong@alderhey.nhs.uk; 8 Department of Women’s & Children’s Health, Institute of Translational Medicine, University of Liverpool, Liverpool L69 3BX, UK

**Keywords:** childhood, paediatric, COVID-19, PIMS-TS, MIS-C, treatment, inflammation, SARS-CoV-2, coronavirus

## Abstract

The novel Severe Acute Respiratory Syndrome Coronavirus 2 (SARS-CoV-2) is the pathogen responsible for Coronavirus Disease 2019 (COVID-19). Whilst most children and young people develop mild symptoms, recent reports suggest a novel paediatric inflammatory multisystem syndrome temporally associated with SARS-CoV-2 (PIMS-TS). Case definition and classification are preliminary, treatment is empiric and disease-associated outcomes are unclear. Here, we report 29 patients with PIMS-TS who were diagnosed, admitted and treated in the English North West between March and June 2020. Consistent with patterns observed internationally, cases peaked approximately 4 weeks after the initial surge of COVID-19-like symptoms in the UK population. Clinical symptoms included fever (100%), skin rashes (72%), cardiovascular involvement (86%), conjunctivitis (62%) and respiratory involvement (21%). Some patients had clinical features partially resembling Kawasaki disease (KD), toxic shock syndrome and cytokine storm syndrome. Male gender (69%), black, Asian and other minority ethnicities (BAME, 59%) were over-represented. Immune modulating treatment was used in all, including intravenous immunoglobulin (IVIG), corticosteroids and cytokine blockers. Notably, 32% of patients treated with IVIG alone went into remission. The rest required additional treatment, usually corticosteroids, with the exception of two patients who were treated with TNF inhibition and IL-1 blockade, respectively. Another patient received IL-1 inhibition as primary therapy, with associated rapid and sustained remission. Randomized and prospective studies are needed to investigate efficacy and safety of treatment, especially as resources of IVIG may be depleted secondary to high demand during future waves of COVID-19.

## 1. Introduction

Since the advent of the Coronavirus Disease 2019 (COVID-19) pandemic, dominated by respiratory disease and evolution of acute respiratory distress syndrome (ARDS), cardiovascular compromise, excessive systemic inflammation and coagulopathy in adults [1,2,3], several countries affected by the coronavirus disease [4] pandemic have reported an unusually high number of cases of children hospitalized due to a multisystem inflammatory condition, at times requiring intensive care (Table S1) [5,6,7,8,9,10,11,12,13,14,15,16,17,18,19,20,21,22,23,24,25,26,27,28,29,30,31,32,33]. The Royal College of Paediatrics and Child Health (RCPCH) defined a paediatric inflammatory multisystem syndrome temporally associated with SARS-CoV-2 (PIMS-TS) [34], and the Centers for Disease Control and Prevention (CDC) defined a multisystem inflammatory syndrome in children (MIS-C) [35], detailed in Table S2. Patients present febrile, with signs and symptoms reminiscent of systemic inflammatory responses partially resembling Kawasaki disease (KD), cytokine storm (CS) or toxic shock syndrome [36], commonly including a rash, conjunctivitis, abdominal pain and evidence of cardiac inflammation and/or injury [37]. A pathophysiological association with SARS-COV-2 infection has been suggested, as a high proportion of patients are either SARS-CoV-2 positive by polymerase chain reaction (PCR) or seropositive for anti-SARS-CoV-2 IgG antibodies. However, a causal relationship between SARS-CoV-2 and PIMS-TS has not been formally proven [19,38]. To reach a consensus for evidence-based management guidance, diagnostic criteria have been defined (Table S2).

While PIMS-TS shows similarities to KD [39], patients tend to be older (≥5 years of age) and not all fulfil criteria for KD (Table S1). Cardiac involvement appears to be more frequent in PIMS-TS, inotrope requirement tends to be more common and recovery, in most cases, is swift (Table S1, [18,40]). As in KD, inflammatory markers in PIMS-TS are raised, including pro-inflammatory cytokines (such as IL-6), C-reactive protein (CRP), ferritin, lactate dehydrogenase (LDH) and neutrophil counts. D-dimers are elevated and myocardial injury can be associated with elevated troponin T and pro-brain natriuretic peptide (pro-BNP). Lymphopenia [41,42], thrombocytopenia and circulating immune complexes have been reported, all distinct from classical KD [41,43]. Lastly, coagulopathy associated with antiphospholipid antibodies has been repeatedly described in PIMS-TS and COVID-19 [44,45] and contributes to a hypercoagulable state [46]. Therapeutically, the hyperinflammatory state in PIMS-TS and severe COVID-19 has been addressed by a variety of immunomodulatory approaches, including corticosteroids, intravenous immunoglobulins (IVIG), cytokine blocking strategies targeting IL-6 (tocilizumab [47,48]) or IL-1 (anakinra [45,49]) and adjunctive anticoagulant treatments [50].

This study focused on clinical presentations, treatment response and outcomes in a cohort of 29 patients with PIMS-TS diagnosed and treated in the English North West between March and June 2020.

## 2. Patients and Methods

Patients meeting RCPCH criteria for PIMS-TS [34] presenting to Alder Hey Children’s NHS Foundation Trust, Liverpool, UK, and Royal Manchester Children’s Hospital, Manchester, UK, between March and June 2020 were included in this study. The Clinical Audit Department approved the study. The Clinical Audit Department (Institutional Review Board) approved data collection, analysis and dissemination of results, in accordance with the Code for Good Practice (audit registration number 6417).

Demographic, laboratory and clinical data were collected retrospectively from hospital patient charts. Laboratory and clinical parameters underlying significant physiological age-related changes (lymphocyte counts, blood pressure, respiratory rate and heart rate) were adjusted using age-specific normal ranges [51,52,53]. For age adjustment of lymphocyte counts, the patient’s absolute lymphocyte count was divided by the 10th centile of the age-adjusted CD3^+^ lymphocyte count [51] and expressed as a ratio, thus allowing a comparison of age-dependent parameters across different age groups. Relevant clinical and laboratory parameters were collected at admission, at their respective peak, at 48 h after symptom resolution, and at the 1–2 week follow-up visit after patient discharge.

The following case definitions for determination of symptom complexes were used as comparators: the American Heart Association criteria for classic and incomplete KD [54], Ravelli criteria for risk assessment of secondary macrophage activation syndrome [55] and calculation of the H score [56]. Published criteria by the CDC (MIS-C) [35] and the RCPCH (PIMS-TS) [34] were applied (Table S2).

Statistical analysis was performed using SPSS 22.0 (SPSS Inc., Chicago, IL, USA). Quantitative variables were reported as absolute numbers, percentages, means and standard deviations where following normal distribution, and by median and interquartile range (IQR) where not. Comparisons of continuous variables between groups to test for equality were performed using the *t* test and paired *t* test if normally distributed (Shapiro–Wilk, *p* > 0.05), or Mann–Whitney and Kruskal–Wallis test where not. Non-parametric continuous dependent samples in ≥2 groups were compared using the Friedman test. Tests of association between categorical variables were based on Chi Squared- and Fisher exact tests. Where applicable, Holm–Bonferroni correction was performed to correct for multiple comparisons, and the significance level adjusted accordingly as indicated. In all other instances, *p*-values reported are 2-sided and were considered statistically significant if <0.05.

## 3. Results and Discussion

### 3.1. Epidemiology and Demographics

**Figure 1:**
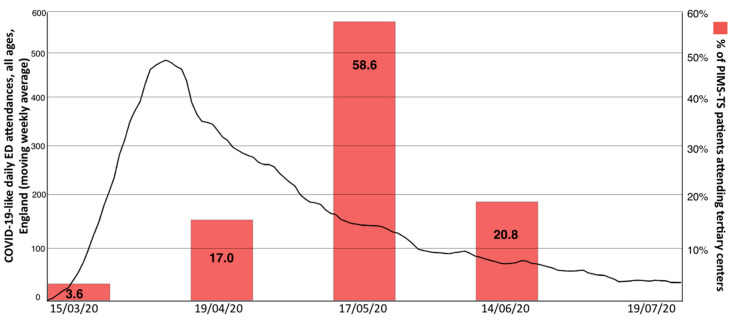
Temporal distribution of paediatric inflammatory multisystem syndrome temporally associated with Severe Acute Respiratory Syndrome Coronavirus 2 (SARS-CoV-2) (PIMS-TS) cases of this cohort, in relation to COVID-19 like presentations to hospitals in England. The peak of presentations of children with paediatric inflammatory multisystem syndrome temporally associated with SARS-CoV-2 followed the peak of presentations of patients, adult and paediatric, to English Emergency Departments, with a lag of 4–6 weeks (Figure adapted from https://www.gov.uk/government/news/weekly-covid-19-surveillance-report-published; week 30).

Between March and June 2020, 29 children were admitted to tertiary paediatric centres in the English North West with a diagnosis of PIMS-TS (Alder Hey Children’s NHS Foundation Trust Hospital, Liverpool, *n* = 10; and Royal Manchester Children’s Hospital, *n* = 19). Notably, this lagged behind the peak of adult admissions for COVID-19 to hospitals in the region by approximately 4 weeks and therefore occurred well into the decline of COVID-19 in England (Figure 1) [57]; in keeping with PIMS-TS/MIS-C cohorts described elsewhere [9].

**Figure 2:**
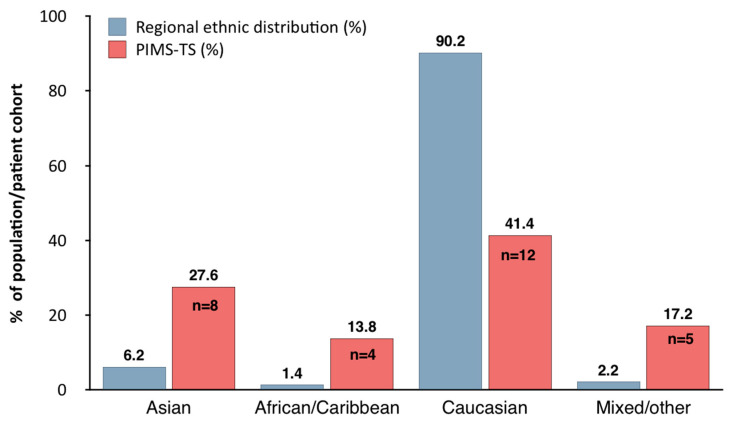
Distribution of ethnicities among children presenting with paediatric multisystem inflammatory syndrome temporally associated with SARS-CoV-2 in relation to regional ethnic distribution in the North West of England, as per National Census Data (https://www.ethnicity-facts-figures.service.gov.uk/uk-population-by-ethnicity/national-and-regional-populations/regional-ethnic-diversity/latest#ethnic-groups-by-area.

Two-thirds of paediatric patients admitted with PIMS-TS (20/29; 69%) were male. Twelve children were Caucasian (41.4%), 6/29 (20.7%) South East Asian, 2 (6.9%) East Asian, 4/29 (13.8%) African/Caribbean and 5 (17.2%) of unknown or multi-ethnic background. In keeping with other published reports from Europe and North America, children of black, Asian and other minority ethnic (BAME) background were over-represented when compared to the composition of the general population in the region based on national census data (Figure 2) [58].

Median age was 6.0 years (IQR 3.8–9.9 years). Only one patient was younger than one year, five between 12 and 24 months, eight 2–5 years, eleven 5–12 years and four older than 12 years of age. On average, patients were hospitalized for 8.5 days (SD 3.1).

### 3.2. Laboratory Evidence of SARS-CoV-2

Half of the patients (14/29; 48.3%) tested positive by SARS-CoV-2 serology, 11 (37.9%) negative. In 13.8% (*n* = 4) no serological testing was undertaken. Of 27 patients tested by SARS-CoV-2 PCR, only three were positive (11.1%). All PCR positive patients were also positive by serology. Combined, 14/29 (48.3%) patients had laboratory evidence of SARS-CoV-2 infection, while population seroprevalence in children in the UK is reported to be much lower, at an adjusted population seroprevalence in England across all ages of 6.0–6.8% [57,59], and even lower among children [60] in who seroprevalence ranges between 0.7 and 10% [61].

**Figure 3:**
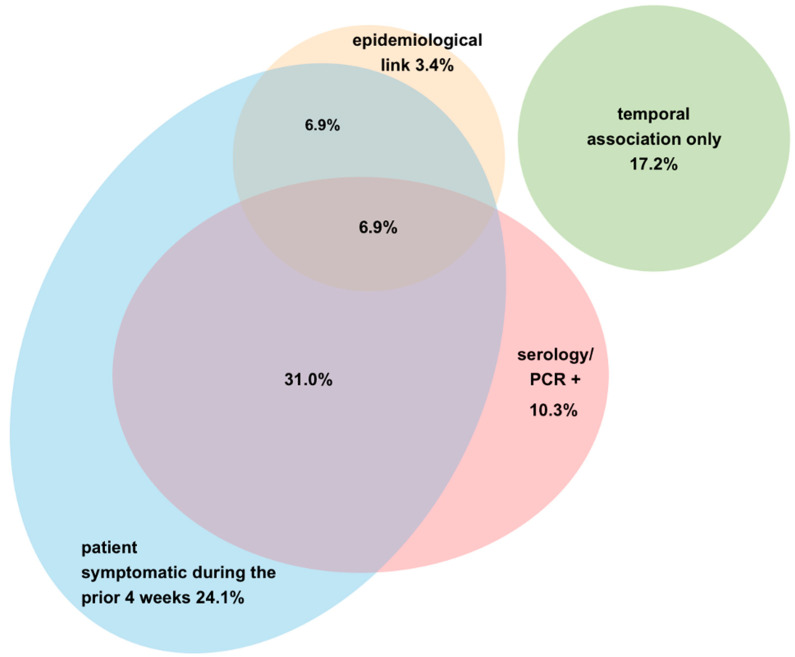
Nature of SARS-CoV-2 exposure to patients included in this cohort.

Of these, one child had PCR-proven SARS-CoV-2 infection in the preceding month, another had had close contact with a proven case, four had contact with a suspected case. As many as 18/29 (62.0%) patients had a history of gastrointestinal illness during the preceding month, 3/29 (10.3%) had a history of respiratory infection. In 8/29 (27.5%) children, no history of a symptomatic illness prior to the onset of PIMS-TS was given. Overall, a laboratory, epidemiological and/or anamnestic link with SARS-CoV-2 was established in 24/29 (82.8%) children (Figure 3).

Males and BAME were over-represented. Both males (male 8/20, female 0/9; *p* = 0.03) and children of BAME background (Caucasian 1/12; BAME 7/17; *p* = 0.04) were significantly more likely to present with clinical signs of hypoperfusion or shock. More severe courses of MIS-C resp. PIMS-TS have repeatedly been seen in non-Caucasian children in Europe, and are being reported internationally [62].

PIMS-TS patients of this cohort exhibited significantly increased seroconversion rates in SARS-CoV-2 infections in the UK or paediatric population, even though lower than reported in other cohorts (Table S1). Comparable proportions, namely 4/11 (36%) of children with, and 7/18 (39%) without symptoms in keeping with classic KD were of the for KD typical age of between 1–5 years of age; and 7/19 (37%) of children who did; and 4/10 (40%) who did not fulfil criteria of either classic or incomplete KD were within the typical age-range. A total of 3/10 children (30%) who tested SARS-CoV-2 sero- and PCR positive, and 3/7 (42.8%) who tested negative (on PCR and/or serology) fulfilled classic or incomplete KD criteria. SARS-CoV-2 status was therefore not discriminatory, as suggested by the current diagnostic criteria in use in the UK and Europe (Table S2).

### 3.3. Clinical Presentation on Admission

Febrile illness was the presenting complaint in all 29 patients. Patients had been febrile for a median of 5 days (IQR 4–7 days) before a diagnosis of PIMS-TS/MIS-C was made. In 18/29 (62%) children, PIMS-TS was the initial working diagnosis, in the remaining 11 (38%), acute infectious causes including urinary tract infections, pneumonia, toxic shock syndrome, bacterial lymphadenitis, viral illness with exanthema or sepsis (*n* = 7); malignancy (1), systemic juvenile idiopathic arthritis (*n* = 1) or appendicitis (*n* = 2) were considered, and initial work up directed accordingly.

Age-adjusted heart rates ranged between the 75–95th centile for age in 4/26 (15.3%), above the 99th centile in 17/26 (65.3%) of patients and unavailable in three. Of note, all but three were febrile at ≥38 °C at the time. Of 22 patients for whom blood pressure [63] was available, three had systolic blood pressures below the age-adjusted normal range [52]. Pulmonary involvement was not uncommon: 6/29 (20.7%) required O2 supplementation.

**Figure 4:**
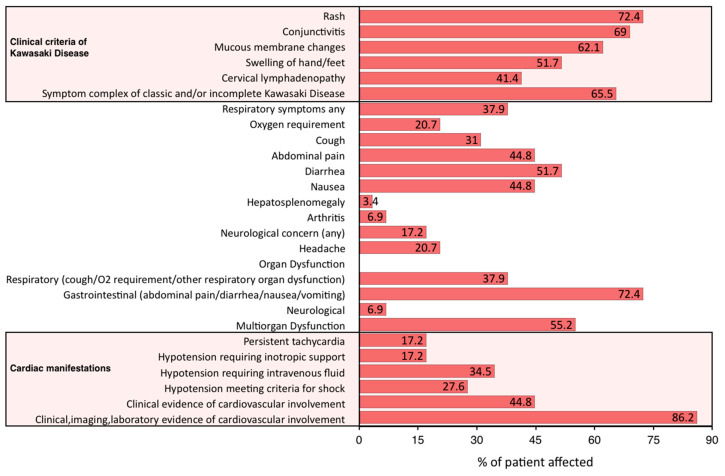
Clinical features of *n* = 29 children presenting with paediatric multisystem inflammatory syndrome temporally associated with SARS-CoV-2 at diagnosis. Patients fulfilling criteria for classic and incomplete Kawasaki Disease and clinical, echocardiographic and laboratory evidence of cardiac injury are separately identified.

Exanthema and gastrointestinal symptoms were common features (Figure 4), in keeping with other published cohorts of PIMS-TS patients, where gastrointestinal symptoms have been reported as common feature, often presenting as the main presenting complaint [9,26] (Table S1).

Except for abdominal pain, which occurred more frequently in children over 5 years of age (3/12 in <5 y.o.a.; 12/17 in ≥5 y.o.a.; *p* = 0.025), clinical features did not differ significantly between age groups. Duration of fever prior to diagnosis did not differ between children with changes on echocardiogram at the 2 week follow-up and those without. Lastly, a composite parameter of cardiovascular involvement at diagnosis (clinical/echocardiographic evidence of functional impairment/laboratory evidence) was neither significantly associated with age nor fulfilment of classical KD criteria.

### 3.4. Laboratory Findings

**Figure 5:**
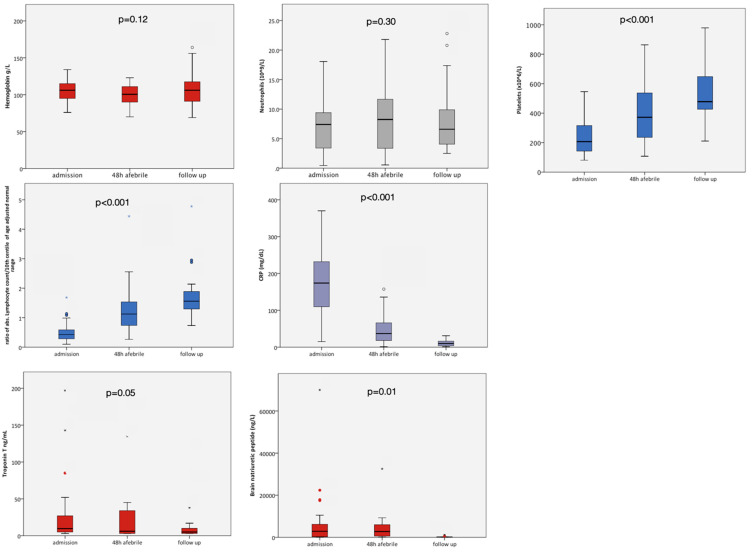
Laboratory parameters at three time points: at diagnosis, at 48 h after the patient being afebrile, at the 1–2 week follow-up, illustrating rapid normalization of thrombocytopenia, lymphopenia, C-reactive protein and brain natriuretic peptide within two weeks of presentation to a health care provider. Lymphocyte count is expressed as a ratio of absolute lymphocyte count divided by 10th centile of normal range for age (see Methods), 1.0 = 10th centile for age adjusted normal range.

Lymphopenia [53] was present in 23/29 (79.3%) patients (Figure 5). In 17 patients, the ratio of absolute lymphocyte count and 10th centile of age adjusted normal range was lower than 0.5, indicating a lymphocyte count of <50% of the age-adjusted 10th centile, affecting the over 5s more prominently (Table 1). By comparison, only one patient had a neutrophil count below, and only 8/29 above the age adjusted normal range at diagnosis. Nine patients presented with thrombocytopenia <150 × 10^9^/L (31%), only one with thrombocytosis ≥500 × 10^9^/L (3.4%).

Acute phase parameters were markedly elevated. Twelve of 19 (63%) children had raised triacylglycerol (TAG) levels at diagnosis (≥150 mg/dL), 20/25 (80%) had ferritin levels above 150 ng/mL with 13/25 (52%) above 500 ng/mL (median 517 ng/mL; IQR 38–10761) (Table S3). All patients exhibited raised CRP levels >8 mg/L (normal range: ≤8 mg/L), 22/29 (75.8%) had CRP levels >100 mg/L (median 174 mg/L; IQR 102.9–232). All but one child had D-Dimer levels above 500 ng/mL, with 18/22 (81.8%) children having D-Dimer levels >1000 ng/mL (normal range ≤500 ng/mL), in 6 cases >5000 ng/mL (27.3%). Serum transaminase levels were elevated in 12/26 (46.2%) children. A subset of patients exhibited mildly raised aPTT ≥33 s (4/19; 21%). International normalized ration (INR) was only marginally elevated in one child at 1.6 (normal 0.8–1.1), and normal in all others. Serum sodium, as a potential symptom of inappropriate anti-diuretic hormone (ADH) release [64], was reduced in a proportion of patients; 17/28 (60.7%) had serum sodium levels ≤135 mmol/L, 5/28 (17.8%) a “very low” sodium of 124–130 mmol/L. No significant association with clinical cardiological outcomes (below) or correlation with blood parameters suggestive of cardiac injury were identified.

While some of these findings also frequently occur in KD, others are uncommon in classical KD (cytopenia, coagulopathy, hypertriglyceridemia) and may help to differentiate between PIMS-TS and KD [64].

### 3.5. Imaging Abnormalities

In the cohort presented here, imaging followed clinical need, but no structured algorithm [65]. Most pathological findings were recorded in cardiac imaging (19/27; 70.4%), including coronary dilatation, valvular regurgitation, functional impairment and pericardial effusions (Table S4).

A total of 21/29 children (72%) underwent chest X-ray or CT, with abnormal findings in half (10/21; 47%). Of note, abnormal chest imaging results were not associated with SARS-CoV-2 seropositivity (*p* = 0.66). Abnormalities identified included perihilar consolidation (4), lymphadenopathy (2), crazy paving pattern (1), ground glass opacities (1) and pleural and pericardial effusion (1). With regard to abdominal imaging, pathological findings were present in 7/19 (36.8%) (Table S5). Whilst gastrointestinal symptoms have been reported as a common complaints in PIMS-TS/MIS-C [16,66,67], the frequency of pathological findings on abdominal imaging in this cohort (36.8%) is striking.

One patient who presented with encephalopathy and headaches underwent a brain MRI and angiogram, which identified focal lesions in the deep white matter, reminiscent of inflammatory lesions seen in acute disseminating encephalomyelitis (ADEM). The patient, who was SARS-CoV-2 seropositive and had no pre-existing comorbidities, responded rapidly to IVMP and IVIG, with complete resolution of neurological signs and symptoms within 48 h. Similar lesions have been reported in PIMS-TS, both in adult and paediatric cases [68,69,70].

End-organ involvement occurs frequently in PIMS-TS, commonly including cardiovascular, gastrointestinal and respiratory involvement; and yield on medical imaging was high.

### 3.6. Classification of Systemic Inflammation

There has been an international focus on classifying patients with PIMS-TS, and early discussions focused on similarities to KD. All patients in this cohort fulfilled RCPCH criteria for PIMS-TS [34], 21/29 North American MIS-C criteria [71] (72.4%), and two thirds of patients criteria for KD [54]. Neither classical nor incomplete KD criteria were more frequent in children <5 y.o.a. when compared to older children (5/12 in <5 y.o.a.; 6/17 in ≥5 y.o.a.; *p* = 1.0).

As a proportion of patients exhibited laboratory (cytopenia, hyperfibrinogenemia, hypertriglyceridemia, etc., see above) and clinical (rash, fevers) features suggestive of CS or macrophage activation syndrome (MAS), we calculated Ravelli scores for secondary MAS. Only one child (15 years, necrotizing lymphadenopathy) fulfilled criteria for MAS [55], (Table 2).

As neither PIMS-TS nor MIS-C criteria are validated at present, their discriminatory value in the diagnosis of this still poorly defined symptom complex remains to be determined. While some fulfilled criteria for classical or incomplete KD, the authors argue that PIMS-TS remains a condition distinct from KD. Some findings, such as lymphopenia and thrombopenia, are present in most PIMS-TS patients, but uncommon in classical KD. Other common symptoms of PIMS-TS/MIS-C are unspecific and can occur during (any viral) infection (including mucocutaneous symptoms, lymphadenopathy). Lastly, cytokine storm (such as sepsis) can be associated with cardiac symptoms, such as coronary dilatation [72].

More specific criteria are required once further data becomes available [50,73], to facilitate an international attempt to classify inflammatory disease associated with COVID-19 in children.

A better understanding of clinical and laboratory features of PIMS-TS may allow preliminary hypotheses and conclusions on its pathophysiology and mechanisms involved. Several mechanisms have been discussed, and include direct immune cell infection and activation, and antibody-dependent enhancement (ADE) [74], a phenomenon shown to contribute to damage accrual during viral infections.

PIMS-TS, severe COVID-19 in adults presents with extensive pulmonary and systemic inflammation, was linked to unfavourable outcomes [75]. Intriguingly, SARS-CoV-2 infection takes mostly a milder course in children [76]. Highest incidence of PIMS-TS occurs weeks after the adult cases of SARS-CoV-2 infection have reached their peak, SARS-CoV-2 PCR is commonly negative, and hence secondary immune events may play a role [77,78,79].

Current criteria as used in Europe are not distinguishing KD and PIMS-TS. Laboratory and clinical differences are now beginning to emerge, which may enable the distinction of these two disorders as separate entities [80]. Both are characterized by systemic inflammation, but differ distinctly with regard to cytokine profiles, lymphocyte subsets and markers of endothelial damage. In the absence of wider availability of these diagnostic opportunities, the discriminatory value of the currently used criteria must be re-evaluated as data becomes available.

A subset of COVID-19 patients reportedly develop vasculitic lesions [43], blood vessel occlusion and infarctions [81,82,83,84]. Indeed, also one of the here reported patients developed IgA vasculitis with renal involvement four weeks after PIMS-TS was diagnosed and treated (see also below).

(Auto-)antibody production, immune complex deposition and mucosal immune dysregulation or poor initial infection control with a delayed immune response, initially high viral loads and delayed or blunted IFN I and III response may cause delayed hyper-inflammatory disease and co-determine the clinical picture of PIMS-TS [77,78,85].

### 3.7. Cardiovascular Involvement

The majority of children included in this study (25/29; 86.2%) exhibited features of cardiac involvement. Thirteen (44.8%) presented with clinical features of cardiovascular compromise, such as poor perfusion, raised lactate or required fluids and/or inotropic support. Elevated biochemical markers of cardiac injury, namely brain natriuretic peptide (BNP) (≥400 pg/mL; 16/22; 72.7%; ≥2000 pg/mL 12/22; 54.5%) or troponin T (≥14 ng/mL; 11/24; 45.6%) were observed.

**Figure 6:**
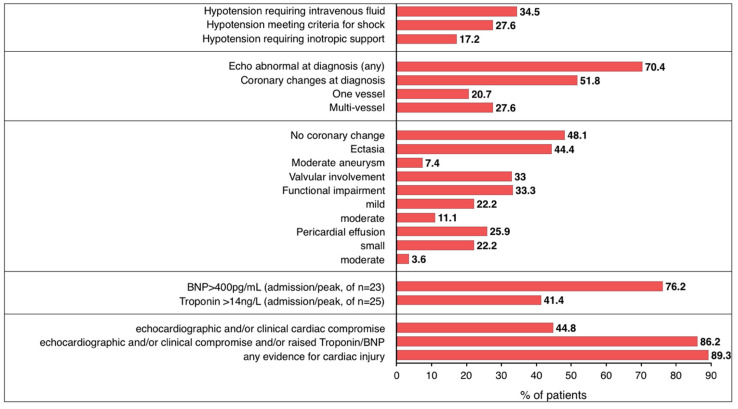
Illustration of clinical, echocardiographic and biochemical evidence of cardiac injury in children with paediatric multisystem inflammatory syndrome temporally associated with SARS-CoV-2. Composite parameters as declared. Troponin—Troponin T, BNP—Brain Natriuretic Peptide. Where data are available for *n* < 29, *n* is specified separately.

Echocardiographic results were retrieved for 27 children (Figure 6, Table S4), abnormalities were documented in 19/27 (70.4%) children.

Common findings included coronary artery changes in 14 patients (51.8%) (Increased echogenicity, dilatation or fusiform aneurysm formation), valvular involvement in 9 (33.3%) and functional impairment in 9 (33.3%) patients. Valvular regurgitation and functional impairment were classified as “mild” in most cases [86].

Neither the number of days febrile before a diagnosis was made (*p* = 0.534), nor patient’s age (*p* = 0.23), were significantly associated with an abnormal echocardiogram on admission (Table S6). BNP (*p* = 0.002) at admission as well as peak BNP (*p* = 0.003) were significantly associated with echocardiographic findings of impaired function, while ferritin, D-Dimers and age-adjusted lymphocyte counts were not.

Need for inotropes (*p* = 0.03) and composite parameters for cardiac injury were more common in those who were seropositive (14/14 SARS-CoV-2 positive had cardiac injury, 6/12 SARS-CoV2 negative had cardiac injury; *p* = 0.004) (Table S6). Clinical features of peripheral hypoperfusion or shock were significantly more common in children with BAME background (*p* = 0.03) and males (*p* = 0.04). Abnormalities on echocardiogram were also more likely to occur in children of BAME background (Caucasian 5/11; BAME 13/14; *p* = 0.02).

Cardiac involvement was the most frequent organ manifestation in this cohort and disproportionately affected children of BAME background.

### 3.8. Treatment and Early Treatment Response

A quarter of patients received inotropic support (7/29; 24.1%), and almost half received fluid boli at presentation (12/29, 41.4%) (Table S7).

Admission levels of BNP but not troponin T were significantly associated with subsequent need for inotropes (Troponin T: inotropes yes: 17 ng/L (IQR 14–42); no: 6.0 ng/L (IQR 3.5–27.0); *p* = 0.06; BNP: inotropes yes: 6180 pg/mL (IQR 3934–46184) no: 965 pg/mL (IQR 233–5183) *p* = 0.02). Peak BNP and troponin T also showed significantly higher levels in children who required inotropes (troponin T inotropes yes 20 ng/L (IQR 14–143); no 5 ng/L (IQR 3.75–31.75) *p* = 0.047, BNP inotropes yes 8902 pg/mL (IQR 3934–62009) no 1264 pg/mL (IQR 236–5183) *p* = 0.008).

As many patients present to local hospitals prior to transfer to a tertiary unit with intensive care facilities, these findings may inform clinical practice, supporting the use of BNP levels in particular as a marker for significant cardiac involvement.

Administration of fluid boluses also was more common in those with higher BNP levels at diagnosis (fluid bolus yes 4615 pg/mL (IQR 965–54018); no 1367 pg/mL (IQR 222–4894) *p* = 0.06). As BNP is released by cardiomyocytes as a result of increased intra-cardiac volume, at least in some patients, this mechanism may have been involved and possibly attenuated cardiac function [87].

Following KD protocols, and in some cases in response to coronary artery anomalies, 22 children (75.9%) received acetylsalicylic acid (ASA), one patient (3.4%) did not, in six (20.6%) this information was not provided.

All patients received immune modulating treatment for PIMS-TS (Table 3), most concomitantly with antimicrobial agents and supportive measures. Reflecting the evolving guidelines and recommendations, treatment decisions were not uniform.

Time from hospitalization to decision to treat ranged from preceding admission to a tertiary centre (treatment commenced at admitting hospital) to 5 days thereafter (median 0 d (on admission), ranging from 48 h prior to 5 days after admission).

Almost all children (28/29; 96.6%) received IVIG (at 2 g/kg/dose); 11/28 (39.3%) one dose, 17/28 (60.7%) two doses. Six patients (6/28; 21.4%) became afebrile after receiving IVIG alone. One was followed with a short course of corticosteroids after IVIG. All remaining patients (23/28; 82.1%) received either (a) combined IVIG and corticosteroids as initial treatment (9/28; 32.1%)) or (b) IVIG alone at first, which did not result in defervescence and therefore subsequently received corticosteroids, either alone (2/28; 7.1%) or in combination with a second IVIG dose (11/28; 39.2%), or a biologic agent (*n* = 1, infliximab; *n* = 1 anakinra). Of 19 children who initially received IVIG alone, only 6 (31.6%) became afebrile. The remaining 13/19 (68.4%) received a second dose of IVIG in combination with steroids, corticosteroids alone or biologic agents (Table 3). Complications attributed to treatment with IVIG were not uncommon and included hypotension (*n* = 1), capillary leak (*n* = 1), respiratory complications (*n* = 2) and meningism (*n* = 3).

Severity of cardiac presentation may have influenced treatment decisions, as all children without cardiac manifestations at diagnosis were initially given IVIG alone (*n* = 4), compared to 10/25 (40%) of those with cardiac manifestations. Though there was a tendency to use corticosteroids at treatment initiation in patients perceived to be more unwell and those with evidence of cardiac injury, symptom resolution in these patients was quicker and echocardiographic outcome at follow-up was not different.

**Figure 7:**
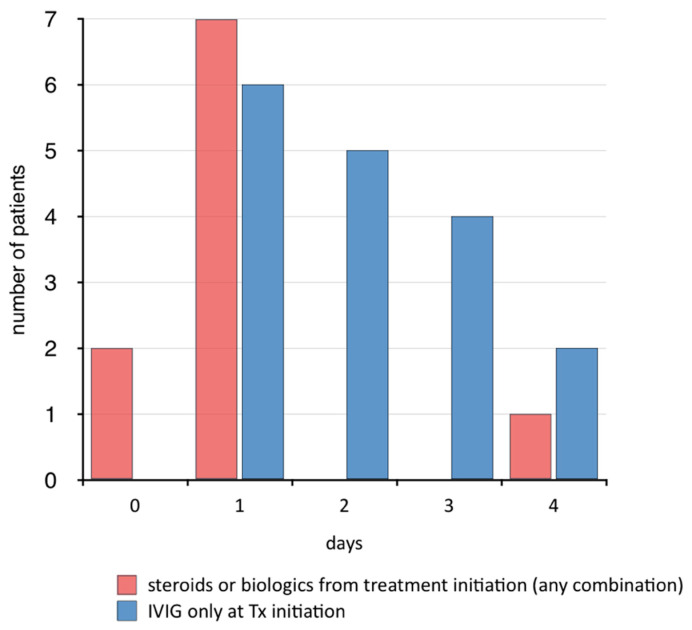
Time from decision to treat to defervescence in days, by treatment regimen, illustrating significantly more rapid achievement of patients becoming afebrile if steroids were included at treatment initiation.

In all but one case, corticosteroids were administered parenterally. Dosing regimens of intravenous Methylprednisolone (IVMP) varied widely. Most patients received IVMP on 3 consecutive days at 10 to 30 mg/kg (max 1 g/d). Following IVMP treatment patients rapidly became afebrile in 20/22 (90.9%). Children on a treatment regimen containing corticosteroids from dose one or biologics [45] became afebrile significantly more rapidly when compared to those who received IVIG alone (median 1 versus 2 days, *p* = 0.015); many of which thereafter required a second dose which was mostly accompanied by steroids (Figure 7).

Though treatment options, composition of agents and individual responses varied significantly, overall, all patients clinically improved. To be considered with caution based on small sample size, IVIG alone appeared to be less effective as compared to the combination with corticosteroids and/or biologic agents. High demand of IVIG during a (potentially) long-lasting pandemic may deplete stocks, and whether targeted cytokine blockade may be equally or even more effective is currently unknown. Optimal treatment for PIMS-TS is currently being evaluated in large multicentre and international trials [50].

### 3.9. Outcomes and Short-Term Prognosis

Next, we investigated short-term outcomes at 48 h after defervescence and 1–2 week follow-up. All patients remained afebrile (≤37.5 °C) at 48 h after defervescence (mean 36.7, SD 0.52, range: 36–38 °C), with the exception of two patients. One child was a 15-year-old who had fulfilled criteria for MAS and was found to have necrotizing lymphadenopathy, another remained febrile despite two doses of IVIG and after four days of IVMP, and defervesced following infliximab.

Vital signs and laboratory parameters of inflammation, CRP, lymphopenia and markers of cardiac injury all normalized, in most cases within 48 h of defervescence (Figure 5). Patients made a sustained recovery following initial defervescence. Of 16 children at one week, two were again febrile after initial defervescence. They also presented with neutrophilia. One patient, a five year-old who was SARS-CoV2 seronegative, had presented with a high BNP (>20.000 pg/mL). The child represented with a CRP of 180 mg/L, BNP and Troponin T had significantly reduced, but a junctional escape rhythm was noted on ECG. The second patient, a 7 year-old represented febrile with a CRP of 219 mg/L and was noted to have reduced ROM in his right hip which self-resolved. Neither child was re-treated, and recovered within the following week. While not febrile, one patient, a 15 year-old girl, developed IgA vasculitis with purpuric skin lesions and mild renal involvement which was self-resolving.

Overall, 12 children (41%) still had clinical signs and symptoms at the 1–2 week follow-up (Table 4).

To monitor cardiac involvement and assess its prognosis, the “most abnormal” echocardiogram during the admission for each patient was compared to follow-up echocardiogram at 2 weeks. Of 19/27 (65.5%) children with abnormal echocardiograms during admission, at FU, coronary involvement was the most frequent finding (*n* = 14), involving the left coronary artery in six, and both left and right coronaries eight children. Severity was “mild” and ranged from coronary ectasia (*n* = 12) to moderate aneurysms (*n* = 2). Nine children had valve involvement, six mild and three moderate functional impairment. Mild pericardial effusion was present in *n* = 7.8/27 children who had a completely normal admission echocardiogram. Of those, 6 were still normal at FU, one was missing FU and another had delayed development of left coronary ectasia, identified at 2 weeks following presentation (Table S8).

Persistent features at 2 weeks were increased echogenicity of the coronary artery wall, ectasia/moderate aneurysms. Ventricular function and valvular regurgitation had almost resolved within this time frame. CT and MRI of these two children with persistent dilatation of coronary artery equivalent to moderate aneurysm were carried out between 4–6 weeks follow-up. This showed normalized coronary artery caliber, and no evidence of myocardial inflammation or late gadolinium enhancement, which suggests that no scarring occurred. In keeping with these findings, a French case series of four cardiac MRIs in children with MIS-C related myocarditis showed diffuse myocardial edema on T2-STIR sequences and native-T1 mapping, with no evidence of late gadolinium enhancement suggestive of replacement fibrosis or focal necrosis, favouring post-infectious myocarditis in children and adolescents with COVID-19 [88].

In summary, in almost none of the PIMS-TS patients with an abnormal echocardiogram during admission, echocardiographic findings at the 2 week FU had not completely normalized, but half of them showed significant improvement. At the 3–5 months follow-up, echocardiographic features continue to improve with another four now having normal echocardiograms. In only one case, right and left coronary ectasia was newly detected on FU echocardiogram. Cardiac outcomes were not associated with clinical or laboratory features during admission. By 2 weeks of follow-up, the differences in echocardiographic changes seen at presentation with regard to sex and ethnic background were no longer present (abnormalities on echocardiogram at follow-up: male 4/16, female 3/8; *p* = 0.65 and Caucasian 6/10, BAME 11/13; *p* = 0.34).

## 4. Conclusions

While children rarely develop severe symptoms related to SARS-CoV-2 infections, some experience highly inflammatory disease requiring hospital admission and treatment. Current classification (PIMS-TS and/or MIS-C) are of limited diagnostic value as not highly disease specific and, based on the recent emergence of this condition, not clinically validated. Thus, international collaboration is urgently needed to collect clinical information and produce more specific diagnostic and/or classification criteria.

PIMS-TS exhibits key features of CS with raised acute phase reactants, cytopenias and organ involvement of the heart, respiratory and/or gastrointestinal tract. While, in the absence of published laboratory studies, the pathophysiology of PIMS-TS remains unknown, demographic, epidemiologic and clinical data may allow for the development of hypotheses and preliminary conclusions. Based on comparisons with national census data, ethnic distribution shows a noticeable over-representation of minority ethnicities. Whether genetic predisposition for inflammatory disease plays the key role remains currently unknown.

Interestingly, both onset and peak of PIMS-TS was delayed by approximately 4 weeks and occurred well into the decline of COVID-19 in England. This, together with the observation that seropositivity among PIMS-TS patients was notably higher when compared to age-matched patients experiencing mild disease, may suggest that limited early infection control and pathogen clearance in the upper airway may result in virus replication and tissue damage, contributing to tissue damage, antibody production, immune complex deposition and/or antibody-dependent enhancement, which may all trigger pro-inflammatory phenotypes.

Current treatment strategies are empiric and not based on evidence. They include symptomatic and supportive measures, and immune modulating treatment. The here presented data, while recognizing its limitations, may suggest that frequently used IVIG may not be sufficient in all patients, and corticosteroids or cytokine blocking agents may result in more rapid defervescence. Larger cohorts within prospective and controlled trials are needed to test this hypothesis, further emphasizing the importance of clinical trials and international collaboration.

While currently available reports, including this study, suggest mostly favourable outcomes in PIMS-TS/MIS-C, data on medium- to long-term outcomes are not available, and only concerted efforts to achieve a structured, comprehensive and multidisciplinary follow-up will allow evidence-based therapeutic strategies in PIMS/TS.

## Figures and Tables

**Table 1:** Median (interquartile range) of complete blood count results at admission, in children presenting with paediatric inflammatory multisystem syndrome temporally associated with SARS-CoV-2. Included is the age-adjusted lymphocyte count, expressed as a ration of patient’s absolute lymphocyte count divided by the 10th centile of age-adjusted normal lymphocyte count.

Parameter	All (Median; IQR)	<5 Years; *n* = 10	≥5 Years; *n* = 17	*p*
Haemoglobin (g/L)	104.0 (93–115)	105.0 (88.5–110)	102 (93–125.5)	0.47
White blood count (×10^9^/L)	9.7 (4.2–12.6)	9.8 (4.3–18.8)	9.6 (4.0–11.4)	0.53
Neutrophils (×10^9^/L)	7.4 (3.0–10.0)	7.5 (2.5–13.4)	7.1 (3.5–9.2)	0.58
Lymphocyte count (×10^9^/L)	1.1 (0.4–1.5)	1.7 (0.8–3.9)	0.54 (0.36–1.2)	0.001 *
Age-adjusted lymphocyte count (1.0 = 10th centile of age adjusted normal range)	0.43 (0.25–0.62)	0.52 (0.36–0.97)	0.34 (0.2–0.57)	0.04 *
Platelet count (×10^6^/L)	207 (137–339)	260 (145–375)	223.8 (125.9)	0.30

*—statistically significant.

**Table 2:** Correlation of symptoms in children with paediatric multisystem inflammatory syndrome temporally associated with SARS-CoV-2 with symptoms of Kawasaki Disease and macrophage activation syndrome.

Features of Systemic Inflammation	*n*	%
RCPCH PIMS-TS criteria	29	100
CDC MIS-C criteria	21	72.4
Features of Kawasaki Disease	19	65.5
Features of classic Kawasaki Disease	11	37.9
Features of incomplete Kawasaki Disease	8	27.6
Features of Macrophage activation syndrome MAS, H score		
≤95	16	61.5
≤130	8	30.8
≤150	1	3.8
≤220	1	3.8
missing	3	10.3

**Table 3:** Treatment regimens used in this cohort. IVIG—intravenous immunoglobulins, SPT—short Prednisolone taper, MP—Methylprednisolone, FU—follow-up.

Treatment Regimen Resulting in Symptom Resolution	*n*	%
1 dose IVIG	4	13.8
2 doses IVIG	1	3.4
2 doses IVIG followed by po Prednisolone taper	1	3.4
1 dose IVIG comb MP followed by SPT	6	20.7
2 doses IVIG comb MP followed by SPT	3	10.3
IVIG, then IVIG comb MP followed by SPT	9	31.0
1 dose IVIG followed by MP followed by SPT	1	3.4
2 doses IVIG followed by MP followed by SPT	1	3.4
IVIG followed by IVIG comb MP, then Anakinra, followed by SPT	1	3.4
Anakinra	1	3.4
IVIG, followed by IVIG comb MP followed by SPT, then Infliximab	1	3.4
Total	29	100.0

**Table 4:** Clinical features observed in patients with paediatric inflammatory multisystem syndrome temporally associated with SARS-CoV-2 at follow-up 1–2 weeks after discharge. Three children presented with arrhythmias that were identified as a new finding following their admission.

Clinical Complications Observed at 2 Weeks FU:	*n*
Reoccurrence of rash	2
Occurrence of new vasculitic rash	1
Edema of hands and feet	1
Reoccurrence of palmar erythema	1
Hepatomegaly (mild)	1
Abdominal pain	2
Proteinuria	1
Subjective palpitations	1
Junctional arrhythmia	1
Bradycardia with bundle branch block	1
Asymptomatic	17

## Supplementary Materials

The following are available online at https://www.mdpi.com/2077-0383/9/10/3293/s1. Table S1: Summary of published case series with a minimum of five cases of Paediatric Inflammatory Multisystem Syndrome temporally associated with SARS-CoV-2 (PIMS-TS). Brief summary of demographic data, SARS-CoV-2 positive rate, key clinical findings at presentation and treatment., Table S2: Summary and comparison of the case definitions for Paediatric Inflammatory Multisystem Syndrome temporally associated with SARS-CoV-2 (PIMS-TS), Multisystem Inflammatory Syndrome in children (MIS-C) by the Center for Disease Control, US and the World Health Organization, Table S3: Median (and interquartile range) of blood results on admission, in children with Paediatric Inflammatory Multisystem Syndrome temporally associated with SARS-CoV-2, Table S4: Echocardiographic and clinical evidence for cardiovascular involvement in children presenting with Paediatric Inflammatory Multisystem Syndrome temporally associated with SARS-CoV-2, Table S5: Imaging investigation performed on patients with PIMS-TS, and abnormalities identified, Table S6: Association of laboratory parameters with parameters reflecting cardiac injury. Brain Natriuretic peptide (BNP) on admission and peak BNP, and C-reactive protein (CRP) were significantly associated with several parameters, and composite parameters, for cardiac injury, Table S7: Supportive therapies required in children with PIMS-TS, Table S8: Echocardiogram during admission/ at 2 week follow up.
