# Supplementary material for: Presentation, Treatment Response and Short-Term Outcomes in Paediatric Multisystem Inflammatory Syndrome Temporally Associated with SARS-CoV-2 (PIMS-TS)
Source: J Clin Med. 2020 Oct 14;9(10):3293. doi: 10.3390/jcm9103293 (PMC7602286; doi:10.3390/jcm9103293)
Supplement: Supplementary file 1 [file jcm-09-03293-s001.pdf]

Supplementary Table S1.: Summary of published case series with a minimum of five cases of Paediatric Inflammatory Multisystem Syndrome temporally associated with SARS-CoV-2 (PIMS-TS). Brief summary of demographic data, SARS-CoV-2 positive rate, key clinical findings at presentation and treatment.

First author	Number of patients, country	Key clinical characteristics and treatment responses
Feldstein[9]	N=186; US	SY: 40% KD-like presentation, 8% CAAs, 80% ITU, 20% MV, 48% inotrope requirement, 2% mortality<br>TX: 77% IVIG of which 21% got 2nd dose, 49% GCs, 21% biologic agents
Cheung[68]	N=17, New York, US	Median age 8yrs;<br>SY: 82% GI symptoms, 76% shock, 70.5% rash, 53% lip changes; 70.5% lymphopenia<br>TX: 82.3% GCs, 76% IVIG, 6% Tocilizumab<br>FU: 1 week all well and discharged
Rollando-Cruz[69]	N=15, New York, US	SY: 87% Lymphopenia, 49% thrombopenia, 100% raised CRP, IL1 and IL6 raised<br>53% ventilated, one ECMO, 53% inotropic support;<br>TX: 80% Tocilizumab, 1 patient received convalescent plasma
Kaushik[70]	N=33, New York, US	Median age 10yrs, BAME overrepresented.<br>SY: 69% vomiting, 63% decreased EF, 51% inotrope requirement, 15% mechanical ventilation<br>TX: 54% IVIG, 51% GCs, 36% Tocilizumab 36%, All discharged and recovered at FU
Capone[71]	N=33, New York, US	100% laboratory evidence of SARS-CoV-2,<br>SY: 64% KD criteria, 97% GI symptoms, 76% shock, 79% PICU, 76% inotrope support, 18% mechanical ventilation, 58% myocardial dysfunction<br>TX: 100% IVIG, 70% GCs, 24% needed second line biologic. Rapid improvement within a week, 9/19 had still changes that had improved on echo at d/c
Lee[72]	N=28, Boston, US	Median age 9yrs<br>SY: 25% KD features (complete/incomplete), 38% myocardial dysfunction, 61% PICU, 25% inotropic support<br>EF lower in MIS-C than historical KD cohort,<br>lymphopenia due to CD4+ and CD8+ and NK reduction, low WBC, thrombocytopenia.<br>21% CAA develop in those with few or no KD features,<br>IL6, IL10, sILR2, ferritin elevated but less pronounced than in historical MAS cohort
Miller[42]	N=44, New York, US	97% SARS-CoV-2 seropositivity<br>SY: GI 84%, rash 70%, n=1 terminal ileitis, n=1 thickened bowel loops<br>TX: 95%GCs, 81%IVIG, 18%Anakinra<br>FU: no deaths
Verdoni[5]	n=10, Italy (Province of Bergamo)	Median age 7.5 yrs, 80% SARS-CoV-2 seropositivity<br>80% cardiac involvement, 50% KD shock syndrome, MAS criteria fulfilled 50%,<br>TX: IVIG resistance and need foe GCs 80%<br>comparison with historical KD cohort n=19: PIMS-TS cases older, higher frequency of cardiac involvement and MAS like features.
Pouletty[73]	N=16; Paris, France	Median age 10yrs; 69% SARS-CoV2 positive (serology and PCR)<br>SY: 44% myocarditis, 20% CAAs, 25% pericarditis, 100% raised Troponin T and BNP, 56% neurological symptoms, 94% mucocutaneous symptoms, 37% lymphadenopathy, 70% PICU<br>TX: 94% got IVIG, only 31% had Tx success after 1 dose IVIG, 62% got 2nd dose; and/or steroids, biologics. Compared to historical KD: older, more pronounced thrombocytopenia, more frequent myocarditis, more frequent IVIG resistance<br>FU: at 14 days all afebrile, no data on FU echo
Belhadjer[17]	N=35, France, Switzerland	Median age 10yrs, 88% SARSciV2 seropositive<br>SY: 83% gastrointestinal symptoms, 65% respiratory symptoms, 60% lymphadenopathy, 57% rash, 31% meningism,<br>80% shock, 3% arrythmia, no CAAs, frequent myocardial dysfunction on echocardiogram<br>EF: <30%: 28%; 30-50%: 72%

		80% inotropic support, 28% ECMO<br>TX: 100% IVIG, 33% additional steroids,<br>FU: no deaths, rapid recovery of systolic function
Belot[13]	N=156, France	Median age 8yrs<br>SY: 61% KD-like presentation fulfilling criteria, 70% myocarditis, 23% macrophage activation syndrome, 22% serositis, 67% PICU of which 73% inotrope requirement
Grimaud[74]	N=20, France	included: PIMS-TS requiring PICU admissions with myocarditis, 67% SARS CoV2 seropositive<br>SY: Commonly abdominal pain, 95% inotrope requirement, 42% mechanical ventilation, myocarditis much more common than in KD<br>TX: 100% IVIG, 10% GCs, n=1 Anakinra, n=1 Tocilizumab
Toubiana[75]	N=21, France	Median age 7.9yrs, overrepresentation of BAME, 90% seropositive SARS CoV2<br>SY: 57% KD shock syndrome, 76% myocarditis; 24% CAAs, 81% ICU. 100% GI symptoms and high inflammatory markers<br>TX: 100% IVIG, 48% GCs. improvement and discharge after 5-15d
Ramcharan[76]	N=15; UK	Median age 8.8yrs, over-representation of BAME<br>SY: 53% respiratory support required, 67% inotropes required<br>TX: 66% IVIG, of which 20% required second dose. remaining 33% received GCs only with OST<br>FU: at 2 weeks all clinically improved, 51% of those with initially abnormal function normalized echocardiographic appearances. good short term cardiologic outcome
Whittaker[10]	N=58, UK	Median age 9yrs; 26% SARS-CoV-2 PCR positive, 87% SARS CoV2 seropositive<br>SY: 100% fever, 53% abdominal pain, 52% diarrhea, 52% rash, 50% shock, 18% fulfilling KD criteria, 14% CAAs, 47% inotrope requirement<br>TX: 71% IVIG, 64% GCs, n=3 anakinra, n=8 infliximab. 22% no treatment and recovered
Chiotos[77]	N=6, UK	prominent cardiac dysfunction, high Troponin T and BNP, enteropathy and gastrointestinal symptoms more common than KD, thrombocytopenia<br>4/6 had neurological symptoms, n=1 underwent LP and had aseptic meningitis
Davies [78]	N=78, UK (PICU admissions only)	Median age 11yrs, BAME overrepresented. SARSCoV2 seropositive 90%<br>SY: 87% shock , 62% abdominal pain, 63% vomiting, 64% diarrhea, 46% mechanical ventilation, 83% inotrope requirement, 36% CAAs<br>TX: 73% steroids, 76% IVIG, 22% biologic agents<br>ECMO n=3, deaths n=1
Moraleda[79]	N=31, Spain	Median age 7.6 yrs; 97% SARS-CoV2 seropositive<br>SY: 67% rash, 67% mucocutaneous symptoms, 48% shock/hypotension, 80% myocardial dysfunction, 61% CAAs, 87% gastrointestinal symptoms<br>One patient with ALL and Trisomy died
Godfred-Cato[38]	N=570, US, CDC data	SY: 61.9% abdominal pain, 61.8% vomiting, 55.3% skin rash, 53.2% diarrhea, 49.5% hypotension, 48.4% conjunctivitis,<br>90.9% gastrointestinal involvement, 86.5% cardiovascular involvement, 70.9% dermatologic or mucocutaneous involvement, 18.4% acute kidney injury<br>40.6% cardiac dysfunction, 35.4% shock, 22.8% myocarditis, 18.6% coronary artery dilatation or aneurysm,<br>63.9% PICU admission
De Farias[80]	N=11, Brazil	Abnormal echocardiogram 63%,<br>mortality 18% (2)
Mamishi[81]	N=45, Iran	Median age 7yrs<br>SY: 58% abdominal pain, 53% rash, 51% conjunctivitis, 18% myocarditis, 31% CAAs<br>TX: 60% GCs, 48% IVIG, mortality 11%
Torres[82]	N=27, Chile	Median age 6yrs, SARS-CoV-2 seropositivity 80%,<br>SY: 64% gastrointestinal symptoms, 63% diarrhea, 15% cardiac dysfunction, 16% CAAs, 11% pericardial effusion, 60% PICU admission<br>TX: 24/27 received immunomodulatory treatment, 12/27 IVIG and steroids, 7/27 IVIG, 5/27 steroids only, 2/27 Tocilizumab<br>even though some echocardiographic changes emerged early during follow-up, favorable outcome in all
Jain[83]	N=23, India	Median age 7.2 years, SARS-CoV2 seropositivity 30.4%, PCR positive 39.1%

		SY: left ventricular dysfunction 35%, CAAS 26%, Mechanical ventilation 39.1%<br>TX: 65% IVIG, 95% GCs, 13% Tocilizumab
Dhanalakshmi[84]	N=19, India	Median age 6yrs, SARS-CoV2 seropositivity 58%<br>SY: 100% fever, 74% mucocutaneous involvement, 63% cardiovascular involvement, 42% gastrointestinal symptoms, 100% raised inflammatory markers, frequent coagulopathy, 63% PICU admission, 31% inotrope requirement<br>TX: 26% IVIG only, 16% GCs only, 42% IVIG and GCs, n=1 IVIG and Tocilizumab
Pererira[85]	N=6, Brazil	SY: 100% fever and increased inflammatory markers, organ involvement- cardiac 100%, renal 66%, respiratory 66%, hematologic 66%, neurologic 16%<br>myocardial dysfunction 50%, CAAs 50%, KD shock syndrome 33%

SY—symptoms, TX—treatment, KD—Kawasaki disease, CAAs—coronary artery aneurysms, IVIG—intravenous immunoglobulin, GCs—glucocorticoids, BAME—Black, Asian, and Minority Ethnicities

Supplementary Table S2.: Summary and comparison of the case definitions for Paediatric Inflammatory Multisystem Syndrome temporally associated with SARS-CoV-2 (PIMS-TS), Multisystem Inflammatory Syndrome in children (MIS-C) by the Center for Disease Control, US and the World Health Organization.

**Synonym**	**RCPCh/ ECDC: PIMS-TS Paediatric inflammatory multisystem syndrome temporally associated with SARS-CoV2 infection**	**CDC: MIS-C (Multisystem inflammatory syndrome in children)**	**WHO: MIS (Multisystem inflammatory syndrome in children and adolescents temporarily related to COVID-19)**
	https://www.rcpch.ac.uk/sites/default/files/2020-05/COVID-19-Paediatric-multisystem-%20inflammatory%20syndrome-20200501.pdf	https://emergency.cdc.gov/han/2020/han00432.asp	https://www.who.int/news-room/commentaries/detail/multisystem-inflammatory-syndrome-in-children-and-adolescents-with-covid-19
**Age**	child	≤ 21 yrs	0-19 years
**Fever**	persistent fever ≥38.5C	Fever ≥38.0°C for ≥24 hours, or report of subjective fever lasting ≥24 hours	fever ≥ 3 days
**Clinical (and laboratory) characteristics**	**AND** evidence of single or multi-organ dysfunction (shock, cardiac, respiratory, renal, gastrointestinal or neurological disorder) **AND** with additional features (most: oxygen requirement, hypotension, some: abdominal pain, confusion, conjunctivitis, cough, diarrhoea, headache, lymphadenopathy, mucous membrane changes, neck swelling, rash, resp symptoms, sore throat, swollen	**AND**<br>evidence of clinically severe illness requiring hospitalization, with multisystem (≥2) organ involvement (cardiac, renal, respiratory, hematologic, gastrointestinal, dermatologic or neurological)	**AND** two or more of the following:<br>1Rash or bilateral non-purulent conjunctivitis or muco-cutaneous inflammation signs (oral, hands or feet) = *stigmata of (incomplete) Kawasaki syndrome*<br>2 Hypotension or shock.<br>3 Features of myocardial dysfunction, (including ECHO findings or elevated Troponin or NT-proBNP), pericarditis, valvulitis, or coronary abnormalities.

	hands and feet, syncope, vomiting). *This may include children fulfilling full or partial criteria for Kawasaki disease.*		4 Evidence of coagulopathy (by PT, PTT, elevated d-Dimers).<br>5 Acute gastrointestinal problems (diarrhoea, vomiting, or abdominal pain).
**Laboratory evidence of inflammat ion**	**AND**<br>inflammation (abnormal fibrinogen, high CRP, high D-Dimers, high ferritin, hypoalbuminaemia, lymphopenia, neutrophilia in most – normal neutrophils in some) some: acute kidney injury, anaemia, coagulopathy, high IL-10 (if available)*, high IL-6 (if available)*, neutrophilia, proteinuria, raised CK, raised LDH, raised triglycerides, raised troponin, thrombocytopenia, transaminitis	**AND**<br>laboratory evidence of inflammation, including, but not limited to, one or more of the following: CRP, ESR, fibrinogen, procalcitonin, d-dimer, ferritin, LDH, or IL-6, elevated neutrophils, reduced lymphocytes and low albumin	**AND**<br>Elevated markers of inflammation such as ESR, CRP, or procalcitonin.
**Exclusion of other conditions**	**AND**<br>Exclusion of any other microbial cause, including bacterial sepsis, staphylococcal or streptococcal shock syndromes, infections associated with myocarditis such as enterovirus (waiting for results of these investigations should not delay seeking expert advice), absence of potential causative organisms (other than SARS-CoV2)	**AND**<br>No alternative plausible diagnoses	**AND**<br>No other obvious microbial cause of inflammation, including bacterial sepsis, staphylococcal or streptococcal shock syndromes.
**SARS CoV2 exposure status**	**AND** SARS-CoV-2 PCR testing may be positive or negative	**AND**<br>positive for current or recent SARS-CoV-2 infection by RT-PCR, serology, or antigen test; or COVID-19 exposure within the 4 weeks prior to the onset of symptoms	**AND**<br>Evidence of COVID-19 (RT-PCR, antigen test or serology positive), or likely contact with patients with COVID-19.

Supplementary Table S3: Median (and interquartile range) of blood results on admission, in children with Paediatric Inflammatory Multisystem Syndrome temporally associated with SARS-CoV-2.

Parameter	Available in (n)	Normal Range	Median; (IQR) min; max
Ferritin admission (ng/mL)	26	1-5yr ≤99; 5-14yr 13-79; 14-19 yr 5.5-67.4	456 (196-722) 38;10761
Ferritin peak (ng/mL)	26		455.0 (218;774) 38;16.053
CRP admission (mg/L)	29	0-8	174 (102.9-232.0)15;370
CRP peak (mg/L)	27		180 (95.5-232.0) 17.2;520
TAG admission (mg/dl)	20	0-9yr ≤100; 10-19yr ≤180	163.7 (123.9-221.3) 61.9;416
Fibrinogen admission (g/L)	22	1.8-3.5g/L	5.1 (4.1-6.5) 1.9;9.9
D-Dimer admission (ng/mL)	21	<500	2371.0 (1481.5-5131.5) 363;10605
D-Dimer peak (ng/mL)	23		2994 (1886-4810) 634; 20474
ALT admission (iu/L)	28	9-36	40.0 (19.25-97.3) 9;223
ALT peak (iu/L)	26		31.0 (21.5-87.5) 7;227
Na admission (mmol/L)	28	132-145	133 (131-136)124;139
aPTT admission (sec)	20	24.2-30.2	28.4 (25.1-31.9) 19.0;38.9
INR admission	16	0.9-1.2	1.16 (1.0-1.3) 0.9;1.6
Troponin admission (ng/L)	24	0-14	9.65 (5-28) 3;197
Troponin peak (ng/L)	25		10.0 (5-47) 3; 354
BNP admission (pg/mL)	22	≤400ng/L HF unlikely; 400-2000ng/L HF possible, ≥2000ng/L HF highly likely	2862 (267-7261) 63;70.000
BNP peak (pg/mL)	23		2740 (269-8902) 56;70.000

HF—heart failure; CRP—C-reactive protein; TAG—triacylglycerol, ALT—alanine transaminase, Na—serum sodium, aPTT—activated prothrombin time.

Supplementary Table S4: Echocardiographic and clinical evidence for cardiovascular involvement in children presenting with Paediatric Inflammatory Multisystem Syndrome temporally associated with SARS-CoV-2.

At diagnosis	*n*	%
Echo abnormal at diagnosis	19/27	70.4
Coronary changes at diagnosis	14/27	51.8
One vessel	6	20.7
Multi-vessel	8	27.6
Coronary changes severity		
Ectasia	12/27	44.4
Moderate aneurysm	2/27	7.4
No coronary change	13/27	48.1
Valvular involvement	9/27	33
Functional impairment	9/27	33.3
mild	6	22.2
moderate	3	11.1
Pericardial effusion	7/27	25.9
small	6	22.2
moderate	1	3.6

Cardiac (cardiac organ dysfunction/inotrope requirement/ shock/ fluid requirement/ Troponin T >14ng/L/ BNP >400pg/mL/ abnormal echocardiogram)	25/29	86.2
Hypotension requiring inotropic support	5/29	17.2
Hypotension requiring intravenous fluid	10/29	34.5
Hypotension meeting criteria for shock	8/29	27.6

Supplementary Table S5: Imaging investigation performed on patients with PIMS-TS, and abnormalities identified.

**Imaging modality**	**Result**	***n*** **(%)**
Abdominal x-ray	Normal	2/2 (100)
Abdominal ultrasound	Normal	4/9 (44%)
	Gallbladder hydrops/ edema	2/9 (22%)
	Splenomegaly	1/9 (11%)
	Lymphadenitis	1/9 (11%)
	Terminal ileitis	1/9 (11%)
	Colitis	1/6 (11%)
Abdominal CT	Normal	3/7 (43%)
	Terminal ileitis	2/7 (29%)
	Colitis	1/7 (14%)
Abdominal MRI	Lymphadenitis	1/1 (100%)
Chest x-ray	Normal	8/21 (38%)
	Consolidation	6/21 (29%)
	Lymphadenopathy	3/21 (14%)
Chest CT	Normal	0/3
	Crazy paving	1/3 (33%)
	Pericardial & pleural effusion	1/3 (33%)
	Consolidation	2/3 (67%)

CT—computertomography; MRI—magnet resonance imaging.

Supplementary Table S6: Association of laboratory parameters with parameters reflecting cardiac injury. Brain Natriuretic peptide (BNP) on admission and peak BNP, and C-reactive protein (CRP) were significantly associated with several parameters, and composite parameters, for cardiac injury.

**Echocardiogram with impaired function at diagnosis**	**yes N (median)**	**no N (median)**	**p**
Age N (mean)	18 (5.6; 3.9)	9 (8.8;4.3)	0.06
BNP admission N (median)	8 (8343)	13 (965)	0.002**
Troponin T admission N (median)	9 (20)	13 (6.0)	0.04
CRP admission N (median)	9 (215)	18 (146.2)	0.04
SARS-CoV2 serology positive	7/8	6/16	0.03
Echocardiogram normal at FU	0/9	7/15	0.02
**Echocardiogram impaired function + clinical compromise (inotrope/fluid bolus/shock) + Trop>14ng/L and/or BNP>400pg/mL at diagnosis**	**yes N (median)**	**no N (median)**	**p**
Age N (mean;SD)	23 (6.5; 4.5)	6 (8.6; 3.9)	0.32
BNP peak (median)	19 (3253)	4 (198)	0.003**
Troponin T peak (median)	21 (15)	4 (5)	0.06
CRP peak (median)	23 (217)	6 (89)	0.02
SARS-CoV2 serology positive	14/20	0/6	0.004**
**Any evidence of cardiac injury (echocardiogram/clinically/laboratory)**	**yes N (median)**	**no N (median)**	**p**
Age N (Mean)	25 (6.7)	4 (8.6)	0.51
BNP peak (median)	21 (2985)	2 (130)	0.03
Troponin T peak (median)	23 (14)	2 (5)	0.37
CRP peak (median)	25 (198)	3 (95)	0.28
SARS-CoV2 serology	14/22	0/3	0.07

Echocardiogram normal at FU	4/21	0/3	0.02

** significant as per Holm-Bonferroni correction.

Supplementary Table S7: Supportive therapies required in children with PIMS-TS.

	n (of total available)	%
PICU admission	6/29	20.7
Suppl. O2 requirement<br>Days suppl. O2	9/24<br>≤3 d: 5<br>4-6d: 4	37.5<br>55.5<br>44.4
Non-invasive ventilation	1/29	3.4
Mechanical ventilation	2/29	6.9
Inotrope requirement<br>Days inotropes:	7/29<br>1 day: 2; 2 days: 2; 6 days: 1<br>uncertain duration	24.1
ECMO	0/29	0
Iv fluids<br>Days iv fluids<br><br>Received fluid bolus<br>Number of boluses	16/29<br>≤3 days: 9<br>4-6 days: 4<br>12/29<br>≤3: 2<br>≤5: 3<br>Unknown number: 7	44.8<br>69.3<br>13.8<br>41.4
Intravenous antibiotics<br>3rd gen Cephalosporin<br>Other beta-lactam<br>Clindamycin	22/24<br>18<br>3<br>2	75.9
Low-molecular heparin	15/22	51.7
Low dose aspirin	22/23	91.3

Supplementary Table S8: Echocardiogram during admission/ at 2 week follow up.

**Echocardiogram at follow up/ at diagnosis**	**normal**	**single vessel ectasia/ aneurysm**	**multiple vessel ectasia/ aneurysm**	**Functional impairment + valve involvement**	**single/multiple vessel ectasia/ aneurysm + valve involvement**	**pericardial effusion**	**missing**
normal	**6**	**1**					**1**
single vessel ectasia/aneurysm		**2**					
single vessel ectasia/aneurysm and functional impairment		**1**					
multiple vessel ectasia/aneurysm		**2**	**2**				**1**
multiple vessel ectasia/aneurysm and functional impairment							
functional impairment with valve involvement				**2**			
single/multiple vessel ectasia/aneurysm and valve involvement	**1**	**1**					

single/multiple vessel ectasia/aneurysm, and valve involvement, and functional impairment		2	2		1		
pericardial effusion						1	1
missing							2

Green—completely normalized, Light green—improved, but not normalized, Light yellow—no change, Red—new abnormal echocardiographic findings at follow up.
